# Intein-mediated split *Sa*Cas9 for genome editing in plants

**DOI:** 10.3389/fgeed.2024.1506468

**Published:** 2025-01-08

**Authors:** Danling Hu, Lizhe Hu, Yaqiang Lu, Xiao Dong, Xingyu Cao, Shasha Bai, Lingang Zhang, Dongming Li, Yongwei Sun

**Affiliations:** Key Laboratory of Herbage and Endemic Crop Biology, Ministry of Education, Inner Mongolia University, Hohhot, Inner Mongolia, China

**Keywords:** CRISPR/Cas, intein, SaCas9, VIGE, plants

## Abstract

Virus-induced genome editing (VIGE) technologies have been developed to address the limitations to plant genome editing, which heavily relies on genetic transformation and regeneration. However, the application of VIGE in plants is hampered by the challenge posed by the size of the commonly used gene editing nucleases, Cas9 and Cas12a. To overcome this challenge, we employed intein-mediated protein splicing to divide the *SaCas9* transcript into two segments (Split-v1) and three segments (Split-v3). The Split-v1 system demonstrated genome editing efficiencies in transgenic plants comparable to those achieved with wild-type SaCas9, with efficiencies ranging from 70.2% to 96.1%. Additionally, we constructed barley stripe mosaic virus (BSMV)-based vectors to co-express Split-v1 SaCas9 and gRNAs targeting *LcHRC*, *LcGW2*, and *LcTB1* in sheepgrass (*Leymus chinensis*), a Gramineae forage species known for its recalcitrance to genetic transformation. Infected leaves of sheepgrass exhibited genome editing efficiencies ranging from 10.40% to 37.03%. These results demonstrate the potential of intein-mediated split nuclease systems to broaden the applicability of VIGE in challenging plant species.

## Introduction

The CRISPR/Cas technology of genome editing is a powerful tool for making targeted changes in plants. However, due to its reliance on genetic transformation and plant regeneration, this technology has been restricted to a handful of species, and even within a species, some varieties are recalcitrant (Zhan et al., 2021). In recent years, virus-induced genome editing (VIGE) technologies have been developed to overcome the limitations to *in vitro* tissue culture and regeneration (Ellison et al., 2020; Liu and Zhang, 2020; Ma et al., 2020; Li et al., 2021; Liu et al., 2024). In VIGE, the modified viral vectors used to express genome editing components were delivered into the plant by agroinfiltration or viral homogenate inoculation, which spread throughout the plant, including the infected meristematic region, and generate heritable gene-edited seeds (Ellison et al., 2020; Li et al., 2021; Lei et al., 2022; Kim et al., 2023). Furthermore, VIGE avoids the stable integration of genome editing components such as expression cassettes of Cas and gRNA (usually seen in biolistics or *Agrobacterium-*mediated transformation) and reduces off-target effects and insertional mutations (Ali et al., 2018). However, because the length of the foreign gene insertion is known to correlate negatively with the stability of the plant virus vector, the size of the two most commonly used gene editing nucleases, Cas9 and Cas12a, poses a challenge for their efficient expression using plant virus vectors, which limits the broad application of VIGE in plants.

Employing a split-protein system has been proven to be an effective method for reducing the size of a gene transcription unit, as demonstrated by several studies (Wright et al., 2015; Kaya et al., 2017). However, the identification of suitable split sites is significantly limited by structural constraints (Wright et al., 2015). Crucially, this strategy has a profound effect on the efficiency of genome editing activities (Costa Carrió, 2015; Payacán Ortiz, 2022). Inteins, which are internal protein elements, facilitate post-translational protein splicing by self-excision from precursor proteins and the ligation of adjacent protein sequences. Recent evidence has shown that intein-mediated splits of *Sp*Cas9 and Cas12j2 are effective in the protoplasts of *Arabidopsis* and rice, respectively (Yuan et al., 2022; Sun Y. et al., 2024). However, there have been no reports yet on the application of intein-mediated Cas nuclease splitting in stable transgenic plants or its potential applicability to VIGE.

In this study, we utilized intein-mediated splicing to split *Staphylococcus aureus* Cas9 (*Sa*Cas9), which comprises only 1,053 amino acids, making it smaller than *Sp*Cas9 (1,386 amino acids), yet it retains a higher efficiency of editing. Results show that an intein-mediated split *Sa*Cas9 system attains editing efficiencies comparable to that of the wild-type *Sa*Cas9 in transgenic plants. We also demonstrate that this intein-mediated split *Sa*Cas9 was successfully applied in VIGE for sheepgrass, a Gramineae forage challenging for genetic transformation.

## Materials and methods

### Vector construction

The full-length SaCas9 was cloned from the SaCas9-gRNA vector reported in Qin et al. (2019). Fragments P1, P2, P3, and P4, fused with intein^N^ or intein^C^, were synthesized by Shenggong Bioengineering (Shanghai). The full-length SaCas9 or fused fragments were cloned into the pCXUN backbone for stable rice transformation or into RNAγ1 or RNAγ2 vectors for VIGE in sheepgrass. The RNAγ1 and RNAγ2 vectors were constructed following the method described by Cheuk and Houde (2018). All subcloning procedures were carried out using the pEASY-Uni Seamless Cloning and Assembly Kit (TransGen Biotech). The plasmid maps, including full-length SaCas9, split-v1, split-v2, and split-v3, are shown in Supplementary Figure S1. The gRNAs targeting *OsNYC1*, *OsNYC4*, and *OsPDS* were synthesized using overlapping PCR. Taking *OsNYC1* as an example, PCR amplification was performed using the SaCas9-gRNA template with the primer pairs U3pmeF/OsNYC1-T1F and OsNYC1-T1F/U3pmeR, producing two PCR products. These products were mixed in a 1:1 ratio and used as the template for a subsequent amplification with U3pmeF/U3pmeR. The resulting PCR product was then cloned into the *Pme* I restriction site of vectors containing either full-length or split SaCas9. For the gRNAs targeting the *LcHRC*, *LcGW2*, and *LcTB1* genes, taking *LcHRC* as an example, the gRNA scaffold was obtained by amplifying the SaCas9-gRNA template using the primers γ1-LcHRC-T1F/γ1-gRNAR. This scaffold was then seamlessly cloned into the *Apa* I restriction site of the RNAγ1 backbone using the Assembly Kit. All primers used in this study are provided in Supplementary Table S1.

### Rice stable transformation

The stable transformation of rice was conducted following previously published protocols (Hiei et al., 1994; Saha et al., 2006). In brief, the vectors described above were transformed into the *Agrobacterium tumefaciens* strain EHA105 by electroporation. Dehulled seeds of the japonica rice (*Oryza sativa L.*) variety Zhonghua 11 were surface sterilized and cultured on the N6D solid medium to induce callus formation. Rice calli were pre-cultured and inoculated with the *A. tumefaciens* strain EHA105 carrying the recombinant vector. After 3 days of co-cultivation, the calli were washed thoroughly with sterilized water to remove residual bacteria and transferred to the N6-S medium for selection under antibiotic pressure for 2 weeks. Resistant calli were then transferred to the RE-III medium for a 2-week cultivation period and subsequently sub-cultured onto the fresh RE-III medium every 2 weeks to promote regeneration. Regenerated plants were successfully obtained, with a minimum of 18 independent transgenic lines generated for each vector.

### Western blot and immunoprecipitation

The Western blot and immunoprecipitation experimental methods were primarily conducted following previously published protocols (Saha et al., 2006). In brief, the protein extract from wild-type and transgenic rice plants was separated by sodium dodecyl sulfate–polyacrylamide gel electrophoresis (SDS-PAGE) and transferred to a polyvinylidene difluoride (PVDF) membrane (Merck Millipore, IPVH00010). For Western blot and immunoprecipitation, *Sa*Cas9 was assayed using the mouse anti-SaCas9 monoclonal antibody [6H4] (EpiGentek, A-9001). The loading control was probed using the anti-actin antibody (Abmart, M20009), with a goat anti-mouse antibody (Abcam, ab6789) utilized as the secondary antibody.

### Plant infection

The constructed plasmids were transformed into *A. tumefaciens* EHA105 strains for agroinfiltration of wild-type or overexpressed RNAα and RNAβ *Nicotiana benthamiana* leaves. Equal volumes of *Agrobacterium* strains harboring individual plasmids were mixed to a final OD_600_ of 0.3 and infiltrated into leaves of 3- to 4-week-old *N. benthamiana* plants, as described previously (Yuan et al., 2011). Virus-infected leaves were harvested after 7 days postinfection (dpi) and ground in 10 mM phosphate buffer each containing 0.5% of celite 545 (Roth) on ice. The virus was used to inoculate the fully emerged third leaves of 2- to 3-week-old sheepgrass using the finger-rub method. Each treatment included a minimum of four replicates.

### Mutagenesis analysis

The mutation types of the transgenic rice plant were assayed by Sanger sequencing of the PCR product and analyzed by CRISPR-GE DSDecodeM software (http://skl.scau.edu.cn/.). For next-generation sequencing (NGS), mutations were analyzed by the online tool high-throughput tracking of mutations 2.0 (Hi-TOM 2.0) (Sun T. et al., 2024). In brief, the target region was amplified from genomic DNA using site-specific primers containing barcode sequences. The resulting amplicons were submitted for next-generation sequencing at the State Key Laboratory of Rice Biology and Breeding, utilizing the Hi-TOM platform (China National Rice Research Institute, Chinese Academy of Agricultural Sciences, Hangzhou).

### Data analysis

Statistical analysis was performed using GraphPad Prism version 8 for Windows (https://www.graphpad.com/), while the figures were further processed with Adobe Illustrator 2020 and Adobe Photoshop CC 2019 (https://www.adobe.com/).

## Results and discussion

### Intein-mediated split *Sa*Cas9 for genome editing in stable transgenic plants

**FIGURE 1:**
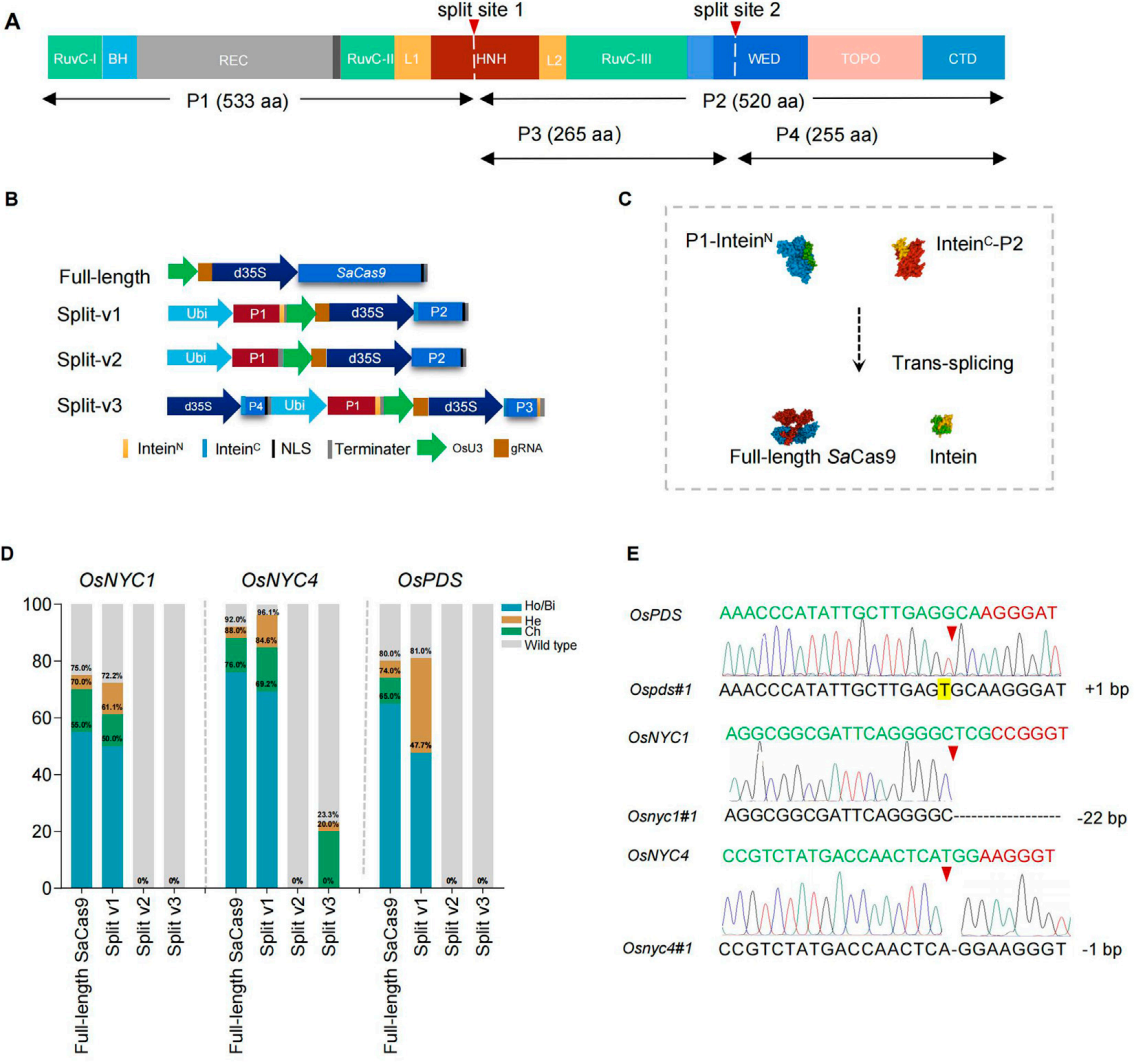
Intein-mediated split SaCas9 for genome editing in plants. **(A)** Domain organization and split sites of SaCas9, with the white dashed line indicating the split site. **(B)** Schematic representation of the split SaCas9 and gRNA expression cassettes. **(C)** Trans-splicing reaction mechanism of split SaCas9 mediated by NpuDnaE intein. **(D)** Mutation types and editing efficiency induced by full-length and split SaCas9 in transgenic rice plants. Ho/Bi indicates homozygous/biallelic mutations; He, heterozygous mutations; Ch, chimeric mutations. **(E)** Sanger sequencing results of regenerated rice plants, with red arrows indicating the edited site. The PAM sequence is highlighted in red, and the protospacer target, in green. Insertions are shaded, and black dashes represent deletions.

**FIGURE 2:**
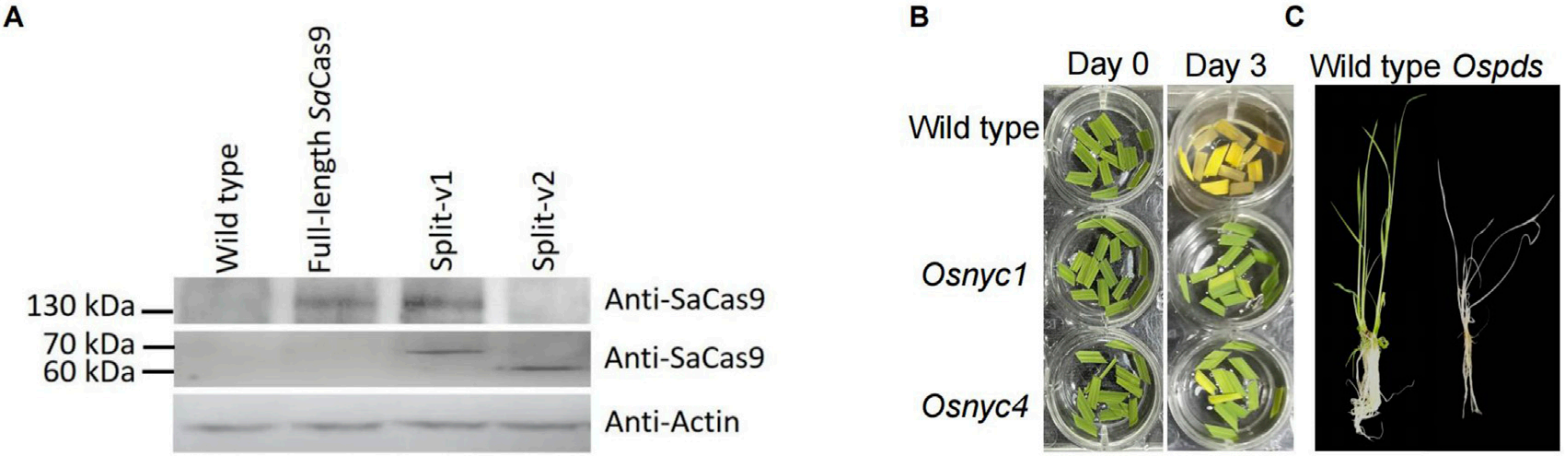
Analysis of SaCas9 trans-splicing in transgenic plants and phenotypes of Ospds, Osnycl, and Osnyc4 mutants. **(A)** Western blot and immunoprecipitation analysis of trans-splicing in SaCas9 protein. Full-length SaCas9 protein is detectable in both full-length SaCas9 and Split-v1 transgenic plants but not in Split-v2. The split SaCas9 protein is detected in both Split-v1 and Split-v2 transgenic plants but not in full-length SaCas9. **(B)** Phenotypes of the Osnycl and Osnyc4 mutants, which exhibit a stay-green phenotype in dark-induced leaf senescence compared to the wild type. **(C)** Phenotypes of the wild-type and Ospds mutant, with photobleaching observed in the Ospds mutant.

To test whether intein-mediated protein splicing is an optional approach to reduce the size of *Sa*Cas9 transcription unit in plants and its potential use in VIGE, we analyzed the sequence of *Sa*Cas9, and results showed that there are 105 potential sites that can be split by intein derived from *Npu*DnaE (Supplementary Figure S2). In this study, the SaCas9 protein was first split into a 533 aa N-terminal fragment (P1) and a 520 aa C-terminal fragment (P2), with the split site located at HNH endonuclease domains that cleave the DNA strands complementary to the guide RNA (split-v1), and these two fragments were fused with N-terminal and C-terminal of *Npu*DnaE intein, respectively (Figure 1 and Supplementary note S1). The fused coding sequence was driven by maize ubiquitin and the double 35 S promoter, respectively (Figure 1B and Supplementary note S1). The full-length *Sa*Cas9 and split *Sa*Cas9 without fusion with intein (split-v2) were designed as control groups for comparison (Figure 1B and Supplementary note S1). Subsequently, three gRNAs driven by an *Os*U3 promoter targeted to the *OsPDS*, *OsNYC1*, and *OsNYC4* genes were constructed and cloned into the vector containing the full-length or split *Sa*Cas9 expression cassette, respectively (Figure 1B and Supplementary note S1). Sequence analysis of the transgenic rice plants revealed that the editing efficiency of split-v1 is comparable to that of full-length SaCas9 across all three target genes. Specifically, the editing efficiencies of split-v1 in *OsNYC1*, *OsNYC4*, and *OsPDS* were 72.2%, 96.1%, and 81%, respectively, while the corresponding efficiencies for full-length SaCas9 were 75%, 92%, and 80%. For the *OsNYC4* and *OsPDS* targets, the editing efficiency of split-v1 was slightly higher than that of full-length SaCas9; however, the proportion of homologous and biallelic mutations was lower, with split-v1 achieving 69.2% and 47.7% for *OsNYC4* and *OsPDS*, respectively, compared to 76% and 65% for full-length SaCas9 (Figures 1D, E). However, split-v2 showed no editing activity (Figrure 1D). These findings are in line with the Western blot and immunoprecipitation results: the full-length *Sa*Cas9 was detected in split-v1 transgenic plants but not in those with split-v2. Possibly due to antibody characteristics or the lower abundance of the target protein, full-length *Sa*Cas9 could not be detected via Western blot, but it can be detected using the immunoprecipitation method (Figure 2A).

To explore the possibility of splitting *Sa*Cas9 into three parts, we divided P2 into P3 and P4 and fused them with intein^N^ or intein^C^ (split-v3), respectively (Figures 1A, B). The results demonstrated a significant decrease in efficiency, with only the vector targeted to *OsNYC4* showing an editing efficiency of 23.3%, and among these edited plants, no homozygous/biallelic lines were found (Figure 1D). The lower efficiency observed in split-v3 may be attributed to the misassembly of P1, P3, and P4, which could potentially be improved using different inteins.

The phenotype of randomly selected *Ospds*, *Osnyc1*, and *Osnyc4* mutants confirmed the sequencing results, with photobleaching observed in the *Ospds* mutant (Figure 2B) and a stay-green phenotype in dark-induced leaf senescence of *Osnyc1* and *Osnyc4* mutants (Figure 2C).

### Validation of intein-mediated split *Sa*Cas9 in VIGE for genome editing of sheepgrass

**FIGURE 3:**
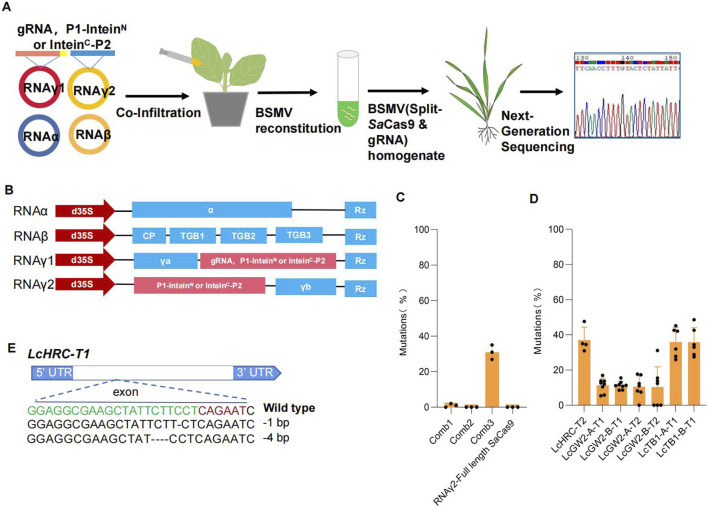
Validation of intein-mediated split-SaCas9 in VIGE for genome editing of sheepgrass. **(A)** Schematic illustration of BSMV-mediated genome editing in sheepgrass. **(B)** Schematic illustration of the vectors used by BSMV-mediated genome editing. **(C)** Genome editing efficiency of different vector combinations targeting the LCHRC gene. **(D)** Genome editing efficiency mediated by BSMV at seven endogenous loci in LCHRC, LCGW2, and LCTB1 genes. For **(C, D)**, each point represents a biological replicate from an independent experiment (n > 3). Data are presented as mean values ±SD. **(E)** Alignment of indel mutations of the LCHRC gene mediated by VIGE. PAM is shown in red, and the protospacer target, in green; black dashes denote deletions.

To test whether split *Sa*Cas9 can be utilized in VIGE, we modified the barley stripe mosaic virus (BSMV) vector as previously described (Cheuk and Houde, 2018). The modified vectors have the four-component BSMV system and have been demonstrated to allow overexpression of cDNAs of up to 2,100 nucleotides. In this study, we utilized RNAγ1 to express a gRNA targeting the *LcHRC* gene of sheepgrass (*Leymus chinensis*). Specifically, we constructed three vector combinations: in the first combination (Comb1), RNAγ1 expressed P1-intein^N^ and intein^C^-P2; in the second combination (Comb2), RNAγ1 expressed P1-intein^N^, while RNAγ2 expressed intein^C^-P2; and in the third combination (Comb3), RNAγ2 expressed both P1-intein^N^ and intein^C^-P2 (Figure 3). These constructs were then introduced into *N. benthamiana* plants for further analysis. After 7 days, the homogenate containing BSMV was harvested and used to infect sheepgrass (Figures 3A, B). After 14 days, we sequenced the *LcHRC* gene of the infected leaves and new leaves using next-generation sequencing. The results show that no editing events were detected in the sheepgrass. We hypothesize that the lack of genome editing activity may be attributed to the excessive amount of *Agrobacterium* components simultaneously injected into *N. benthamiana*. To test this, we separately introduced three vector combinations into *N. benthamiana* plants overexpressing RNAα and RNAβ and subsequently used the resulting virus to infect sheepgrass leaves. Next-generation sequencing revealed detectable genome editing activity in the Comb3-infected leaves, with an average editing efficiency reaching 30.95% (Figures 3B–D). In contrast, genome editing activity was undetectable in the leaves infected with Comb1 and Comb2, as well as in the control group, where RNAγ1 was used to express the gRNA and RNAγ2 was used to express the full-length SaCas9. To evaluate the efficiency of this approach and its applicability as a high-throughput gRNA screening platform in sheepgrass, additional target sites were designed for validation. These included LcHRC-T1 targeting the *LcHRC* gene; LcGW2-A-T1, LcGW2-B-T1, LcGW2-A-T2, and LcGW2-B-T2 targeting the *LcGW2* gene; and LcTB1-A-T1 and LcTB1-B-T1 targeting the *LcTB1* gene. All target sites exhibited detectable editing activity, with genome editing efficiencies ranging from 10.40% to 37.03%. Surprisingly, genome editing activity was not detectable in the new leaves of plants that had not been infected with the virus. This may be due to the size of the split *Sa*Cas9 still having some impact on the pathogenicity or mobility of the virus. Overcoming this limitation can be achieved by 1) optimizing viral vectors to enhance the ability of viruses to express exogenous genes and 2) improving the gene editing efficiency of reported smaller nucleases like CasΦ (Pausch et al., 2020; Liu et al., 2022; Li et al., 2023; Wang et al., 2023; Sun Y. et al., 2024; Zhao et al., 2024), Cas12f1 (Bigelyte et al., 2021; Wu et al., 2021; Kim et al., 2022), Casλ (Al-Shayeb et al., 2022), and TnpB (Karvelis et al., 2021) in plants and employing inteins to facilitate splicing and achieve smaller transcription units.

## Conclusion

We have demonstrated that intein-mediated split *Sa*Cas9 functions effectively in transgenic plants. Additionally, we validated that intein-mediated split *Sa*Cas9 can be successfully applied in the VIGE in sheepgrass, a type of Gramineae forage that is difficult to be genetically transformed. This strategy creates new opportunities not only for VIGE but also for virus-mediated base editing and prime editing in plants.

## Data Availability

The raw data supporting the conclusions of this article will be made available by the authors, without undue reservation.
