# Supplementary material for: Intein-mediated split SaCas9 for genome editing in plants
Source: Front Genome Ed. 2025 Jan 8;6:1506468. doi: 10.3389/fgeed.2024.1506468 (PMC11750852; doi:10.3389/fgeed.2024.1506468)
Supplement: Supplementary file 1 [file DataSheet1.docx]

**Supplementary Materials**

**Intein-mediated split *Sa*Cas9 for genome editing in plants**

Lizhe Hu^†^, Danling Hu^†^, Yaqiang Lu, Xiao Dong, Xingyu Cao, Shasha Bai, Lingang Zhang, Dongming Li^*^ and Yongwei Sun^*^

Key Laboratory of Herbage and Endemic Crop Biology, Ministry of Education, Inner Mongolia University, Hohhot, Inner Mongolia 010070, China.

*Corresponding (Emails: [sunyongwei@imu.edu.cn](mailto:sunyongwei@imu.edu.cn) (YS); lidongming0118@163.com (DL)[;](mailto:qizhi@imu.edu.cn(ZQ);) [)](mailto:lidongming0118@163.com(DL)))

**Supplementary Figure 1. Plasmid maps of full-length SaCas9, Split-v1, Split-v2, and Split-v3.**


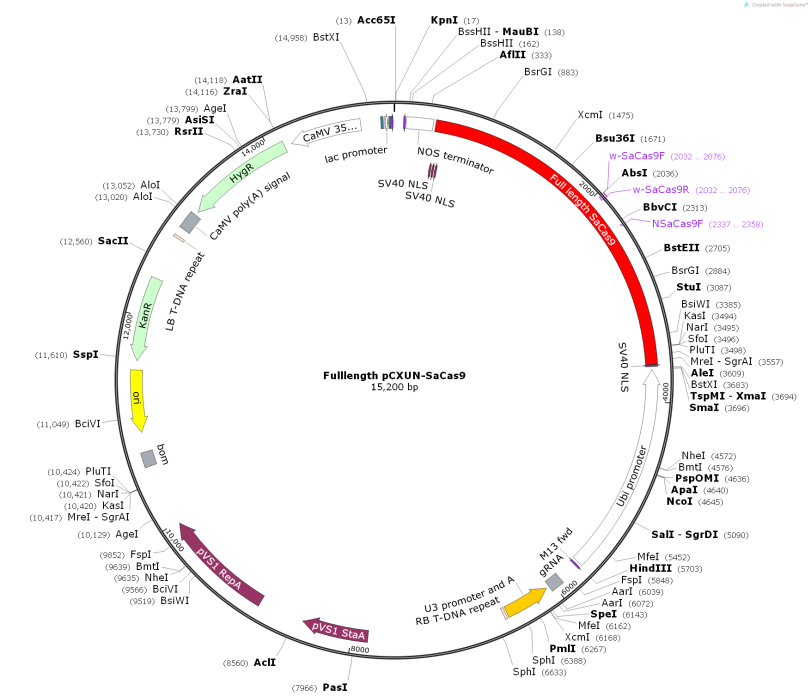


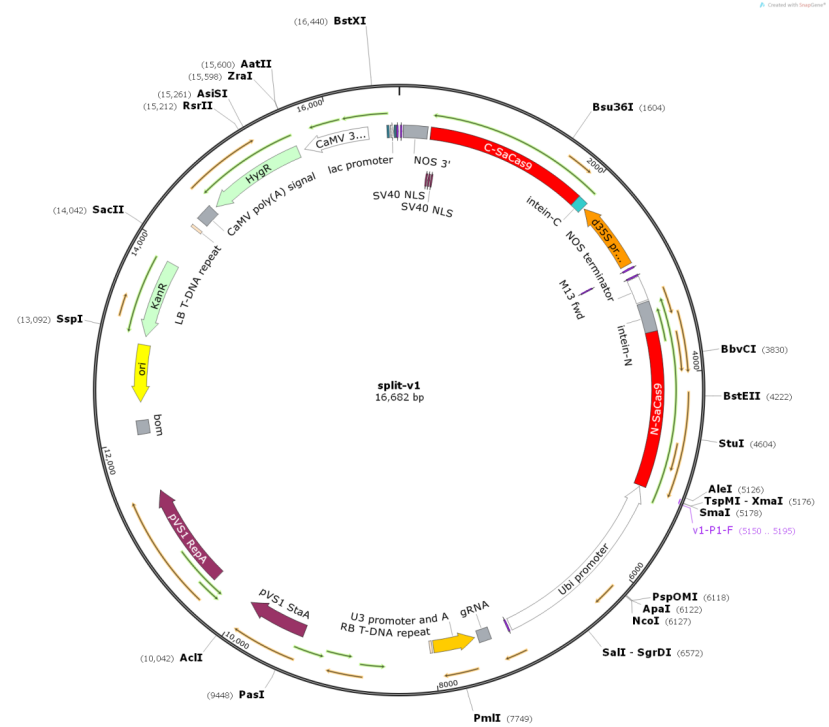


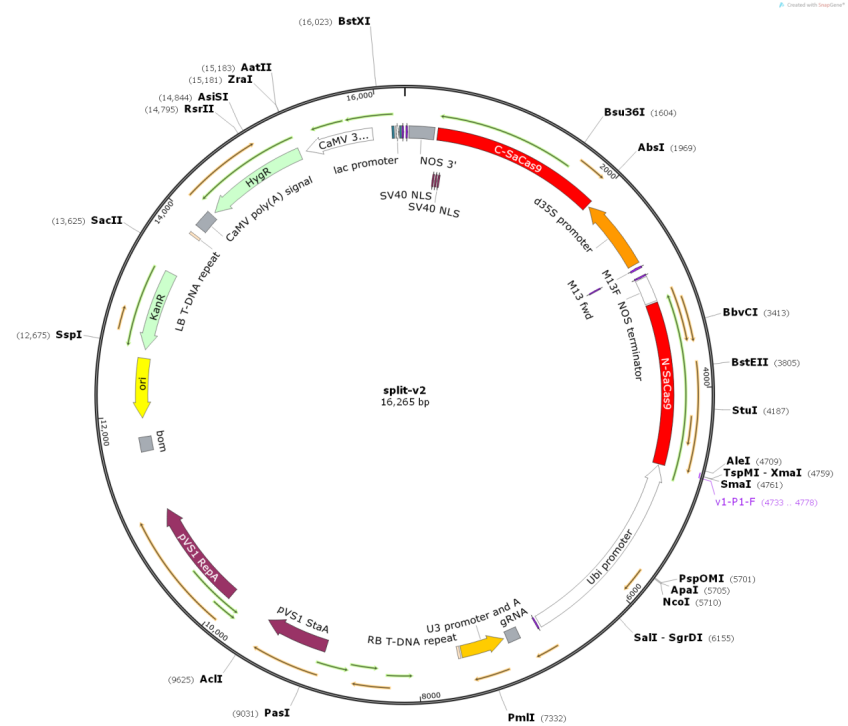

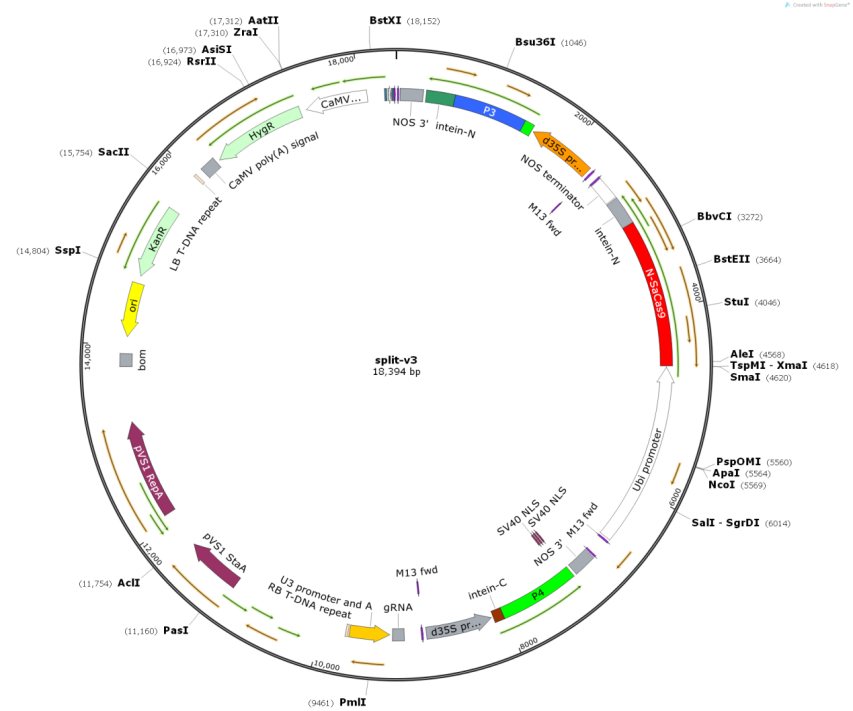


MKRNYILGLDIGITSVGYGIIDYETRDVIDAGVRLFKEANVENNEGRRSKRGARRLKRRRRHRIQRVKKLLFDYNLLTDHSELSGINPYEARVKGLSQKLSEEEFSAALLHLAKRRGVHNVNEVEEDTGNELSTKEQISRNSKALEEKYVAELQLERLKKDGEVRGSINRFKTSDYVKEAKQLLKVQKAYHQLDQSFIDTYIDLLETRRTYYEGPGEGSPFGWKDIKEWYEMLMGHCTYFPEELRSVKYAYNADLYNALNDLNNLVITRDENEKLEYYEKFQIIENVFKQKKKPTLKQIAKEILVNEEDIKGYRVTSTGKPEFTNLKVYHDIKDITARKEIIENAELLDQIAKILTIYQSSEDIQEELTNLNSELTQEEIEQISNLKGYTGTHNLSLKAINLILDELWHTNDNQIAIFNRLKLVPKKVDLSQQKEIPTTLVDDFILSPVVKRSFIQSIKVINAIIKKYGLPNDIIIELAREKNSKDAQKMINEMQKRNRQTNERIEEIIRTTGKENAKYLIEKIKLHDMQEGKCLYSLEAIPLEDLLNNPFNYEVDHIIPRSVSFDNSFNNKVLVKQEENSKKGNRTPFQYLSSSDSKISYETFKKHILNLAKGKGRISKTKKEYLLEERDINRFSVQKDFINRNLVDTRYATRGLMNLLRSYFRVNNLDVKVKSINGGFTSFLRRKWKFKKERNKGYKHHAEDALIIANADFIFKEWKKLDKAKKVMENQMFEEKQAESMPEIETEQEYKEIFITPHQIKHIKDFKDYKYSHRVDKKPNRELINDTLYSTRKDDKGNTLIVNNLNGLYDKDNDKLKKLINKSPEKLLMYHHDPQTYQKLKLIMEQYGDEKNPLYKYYEETGNYLTKYSKKDNGPVIKKIKYYGNKLNAHLDITDDYPNSRNKVVKLSLKPYRFDVYLDNGVYKFVTVKNLDVIKKENYYEVNSKCYEEAKKLKKISNQAEFIASFYNNDLIKINGELYRVIGVNNDLLNRIEVNMIDITYREYLENMNDKRPPRIIKTIASKTQSIKKYSTDILGNLYEVKSKKHPQIIKKG*

**Supplementary Figure 2. The potential split site of *Sa*Cas9 mediated by *Npu*DnaE.**

The potential split site of *Sa*Cas9 mediated by *Npu*DnaE is shadowed. To enhance splicing efficiency, it is essential for the first amino acid in the C-terminal region of proteins to be cysteine, serine, or threonine, shadowed in yellow, green, and pink, respectively. Sometimes, it is also necessary to consider that the cleavage sites should be exposed on the surface. The split site of this study are underlined.

**Supplementary note 1. The sequence of Cas nucleases, gRNA, and intein used in this study.**

**>Amino acid sequence of Intein^N^**

CLSYETEILTVEYGLLPIGKIVEKRIECTVYSVDNNGNIYTQPVAQWHDRGEQEVFEYCLEDGSLIRATKDHKFMTVDGQMLPIDEIFERELDLMRVDNLPN*

**>Amino acid sequence of Intein^C^**

MIKIATRKYLGKQNVYDIGVERDHNFALKNGFIASN

**>Sequence of Ubi-P1-Intein^N^-Nos**

The Ubi-Promoter and intein^N^ were shadowed green and yellow, respectively. The sequence of P1 are in red, the terminator was shadowed grey.

ctgcagtgcagcgtgacccggtcgtgcccctctctagagataatgagcattgcatgtctaagttataaaaaattaccacatattttttttgtcacacttgtttgaagtgcagtttatctatctttatacatatatttaaactttactctacgaataatataatctatagtactacaataatatcagtgttttagagaatcatataaatgaacagttagacatggtctaaaggacaattgagtattttgacaacaggactctacagttttatctttttagtgtgcatgtgttctcctttttttttgcaaatagcttcacctatataatacttcatccattttattagtacatccatttagggtttagggttaatggtttttatagactaatttttttagtacatctattttattctattttagcctctaaattaagaaaactaaaactctattttagtttttttatttaataatttagatataaaatagaataaaataaagtgactaaaaattaaacaaataccctttaagaaattaaaaaaactaaggaaacatttttcttgtttcgagtagataatgccagcctgttaaacgccgtcgacgagtctaacggacaccaaccagcgaaccagcagcgtcgcgtcgggccaagcgaagcagacggcacggcatctctgtcgctgcctctggacccctctcgagagttccgctccaccgttggacttgctccgctgtcggcatccagaaatgcgtggcggagcggcagacgtgagccggcacggcaggcggcctcctcctcctctcacggcacggcagctacgggggattcctttcccaccgctccttcgctttcccttcctcgcccgccgtaataaatagacaccccctccacaccctctttccccaacctcgtgttgttcggagcgcacacacacacaaccagatctcccccaaatccacccgtcggcacctccgcttcaaggtacgccgctcgtcctccccccccccccctctctaccttctctagatcggcgttccggtccatggttagggcccggtagttctacttctgttcatgtttgtgttagatccgtgtttgtgttagatccgtgctgctagcgttcgtacacggatgcgacctgtacgtcagacacgttctgattgctaacttgccagtgtttctctttggggaatcctgggatggctctagccgttccgcagacgggatcgatttcatgattttttttgtttcgttgcatagggtttggtttgcccttttcctttatttcaatatatgccgtgcacttgtttgtcgggtcatcttttcatgcttttttttgtcttggttgtgatgatgtggtctggttgggcggtcgttctagatcggagtagaattctgtttcaaactacctggtggatttattaattttggatctgtatgtgtgtgccatacatattcatagttacgaattgaagatgatggatggaaatatcgatctaggataggtatacatgttgatgcgggttttactgatgcatatacagagatgctttttgttcgcttggttgtgatgatgtggtgtggttgggcggtcgttcattcgttctagatcggagtagaatactgtttcaaactacctggtgtatttattaattttggaactgtatgtgtgtgtcatacatcttcatagttacgagtttaagatggatggaaatatcgatctaggataggtatacatgttgatgtgggttttactgatgcatatacatgatggcatatgcagcatctattcatatgctctaaccttgagtacctatctattataataaacaagtatgttttataattattttgatcttgatatacttggatgatggcatatgcagcagctatatgtggatttttttagccctgccttcatacgctatttatttgcttggtactgtttcttttgtcgatgctcaccctgttgtttggtgttacttctgcagcccgggggatccatgaagcgcaactacatcctggggctggacatcggcatcacctccgtcggctacggcatcatcgactacgagacgcgcgacgtcatcgacgccggcgtccgcctgttcaaggaagccaacgtcgagaacaacgagggccgccgctccaagcgcggcgcccgccgcctgaagcgccgccgccgccaccgcatccagcgcgtcaagaagctgctgttcgactacaacctgctgaccgaccactccgagctgtccggcatcaacccgtacgaggcccgcgtcaagggcctgtcccagaagctgtccgaggaagagttctccgccgccctgctgcacctggccaagcgccgcggcgtccacaacgtcaacgaggtggaagaggacaccggcaacgagctgtccaccaaggagcagatcagccgcaactccaaggccctggaggagaagtacgtcgccgagctgcaactggagcggctgaagaaggacggcgaggtccgcggctccatcaaccgcttcaagacctccgactacgtcaaggaagccaagcagctgctgaaggtccagaaggcctaccaccagctggaccagtccttcatcgacacctacatcgacctgctggagacgcgtcgcacctactacgagggtccgggcgagggctccccgttcggctggaaggacatcaaggagtggtacgagatgctgatgggccactgcacctacttcccggaggagctgcgctccgtcaagtacgcctacaacgccgacctgtacaacgccctgaacgacctgaacaacctggtcatcacccgcgacgagaacgagaagctggagtactacgagaagttccagatcatcgagaacgtgttcaagcagaagaagaagccgaccctgaagcagatcgccaaggagatcctggtcaacgaagaggacatcaagggctaccgcgtcacctccaccggcaagccggagttcaccaacctgaaggtctaccacgacatcaaggacatcaccgcccgcaaggagatcatcgagaacgccgagctgctggaccagatcgccaagatcctgacgatctaccagtcctccgaggacatccaggaagagctgaccaacctgaactccgagctgacccaggaagagatcgagcagatcagcaacctgaagggctacaccggcacccacaacctgtccctgaaggccatcaacctgatcctggacgagctgtggcacaccaacgacaaccagatcgccatcttcaaccgcctgaagctggtcccgaagaaggtggacctgtcccagcagaaggagatcccgaccaccctggtggacgacttcatcctgtccccggtcgtcaagcgctccttcatccagtccatcaaggtcatcaacgccatcatcaagaagtacggcctgccgaacgacatcatcatcgagctggcccgcgagaagaactccaaggacgcccagaagatgatcaacgagatgcagaagcgcaaccgccagaccaacgagcgcatcgaggagatcatccgcaccaccggcaaggagaacgccaagtacctgatcgagaagatcaagctgcacgacatgcaggaaggcaagTGCTTGTCCTACGAGACCGAGATTCTTACTGTTGAGTATGGATTGCTTCCAATCGGCAAGATCGTAGAGAAACGTATTGAGTGTACAGTTTATTCTGTTGACAACAACGGAAATATCTATACTCAGCCTGTTGCTCAATGGCACGATAGAGGGGAACAAGAGGTTTTCGAGTATTGCCTTGAGGATGGTAGCCTCATTCGAGCAACAAAAGATCACAAATTTATGACAGTGGATGGTCAGATGTTACCTATCGATGAAATTTTCGAAAGGGAATTAGACCTCATGAGAGTGGATAACCTCCCTAATTAGccgaatttccccgatcgttcaaacatttggcaataaagtttcttaagattgaatcctgttgccggtcttgcgatgattatcatataatttctgttgaattacgttaagcatgtaataattaacatgtaatgcatgacgttatttatgagatgggtttttatgattagagtcccgcaattatacatttaatacgcgatagaaaacaaaatatagcgcgcaaactaggataaattatcgcgcgcggtgtcatctatgttactagatc

**>Sequence of d35S-Intein^C^-P2-Nos**

The d35S and intein^C^ were shadowed green and yellow, respectively. The sequence of P2 and NLS are in red and green, respectively. The terminator was shadowed grey.

gttgtaaaacgacggccagtgccaagcttgattaccaattcgagctccctacccctactccaaaaatgtcaaagatacagtctcagaagaccaaagggctattgagacttttcaacaaagggtaatttcgggaaacctcctcggattccattgcccagctatctgtcacttcatcgaaaggacagtagaaaaggaaggtggctcctacaaatgccatcattgcgataaaggaaaggctatcattcaagatgcctctgccgacagtggtcccaaagatggacccccacccacgaggagcatcgtggaaaaagaagacgttccaaccacgtcttcaaagcaagtggattgatgtgacatctccactgacgtaagggatgacgcacaatcccacccctactccaaaaatgtcaaagatacagtctcagaagaccaaagggctattgagacttttcaacaaagggtaatttcgggaaacctcctcggattccattgcccagctatctgtcacttcatcgaaaggacagtagaaaaggaaggtggctcctacaaatgccatcattgcgataaaggaaaggctatcattcaagatgcctctgccgacagtggtcccaaagatggacccccacccacgaggagcatcgtggaaaaagaagacgttccaaccacgtcttcaaagcaagtggattgatgtgacatctccactgacgtaagggatgacgcacaatcccactatccttcgcaagacccttcctctatataaggaagttcatttcatttggagaggacagcccagatcactagtATGATCAAGATCGCCACAAGGAAGTACCTCGGCAAGCAGAACGTGTACGATATCGGCGTGGAGCGCGACCACAACTTCGCCCTCAAGAATGGCTTCATCGCCTCCAACTGCCTCTACAGCCTCGAGGCCATCCCGCTCGAGGATCTGCTCAACAACCCGTTCAACTACGAGGTGGACCACATCATCCCACGCTCCGTGTCCTTCGACAACTCTTTCAACAACAAGGTTCTCGTGAAGCAGGAGGAGAATAGCAAGAAGGGCAACAGGACCCCGTTCCAGTACCTCTCCAGCTCCGACTCCAAGATCTCATACGAGACCTTCAAGAAGCACATCCTCAACCTCGCCAAGGGCAAGGGCAGGATCTCTAAGACCAAGAAGGAGTACCTCCTGGAGGAGAGGGACATCAACAGGTTCAGCGTGCAGAAGGATTTCATTAACCGCAACCTGGTGGACACCCGGTACGCGACCAGGGGCTTGATGAACCTCCTCAGGTCCTACTTCCGCGTGAACAACCTGGATGTGAAGGTGAAGTCCATCAACGGCGGCTTCACCAGCTTCCTCCGCCGGAAGTGGAAGTTCAAGAAGGAGCGCAACAAGGGCTACAAGCACCACGCCGAGGATGCCCTGATCATCGCGAACGCCGACTTCATCTTCAAGGAGTGGAAGAAGCTGGACAAGGCCAAGAAAGTGATGGAGAATCAGATGTTCGAGGAGAAGCAGGCCGAGTCAATGCCTGAGATCGAGACCGAGCAGGAGTACAAGGAGATCTTCATCACCCCGCACCAGATCAAGCACATCAAGGACTTCAAGGATTACAAGTACTCCCACAGGGTGGATAAGAAGCCGAATCGCGAGCTGATCAACGATACCCTGTACTCCACACGCAAGGACGACAAGGGCAACACCCTCATCGTGAACAACCTCAACGGCCTCTACGACAAGGACAACGACAAGCTCAAGAAGCTCATCAACAAGTCCCCGGAGAAGCTCCTCATGTACCACCACGATCCGCAGACCTACCAGAAGCTCAAGCTCATCATGGAGCAGTACGGCGATGAGAAGAACCCACTCTACAAGTACTACGAGGAGACCGGCAACTATCTCACCAAGTACTCTAAGAAGGACAACGGCCCAGTGATCAAGAAGATCAAGTACTACGGCAATAAGCTCAACGCCCACCTCGACATCACCGACGATTACCCGAACTCCCGGAACAAGGTGGTCAAGCTGAGCCTGAAGCCGTACAGGTTCGACGTTTACCTCGATAATGGCGTGTACAAGTTCGTGACCGTGAAGAACCTTGATGTGATCAAGAAGGAGAACTACTACGAGGTGAACAGCAAGTGCTACGAGGAGGCCAAGAAGCTGAAGAAGATCAGCAACCAGGCCGAGTTCATCGCGAGCTTCTACAACAACGATCTCATCAAGATCAACGGCGAGCTCTACCGCGTGATCGGCGTCAACAACGATCTCCTCAACCGCATCGAGGTGAACATGATCGACATCACCTACCGCGAGTACCTCGAGAACATGAACGACAAGAGGCCGCCGCGCATCATCAAGACCATCGCCAGCAAGACCCAGTCCATCAAGAAGTACTCCACCGACATCCTCGGCAACCTCTACGAGGTGAAGTCCAAGAAGCACCCGCAGATCATCAAGAAGGGCTCCGGCGGCAGCCCGAAGAAGAAGCGCAAGGTGAGCGGCGGCTCCCCTAAGAAGAAGCGCAAGGTGAGCGGCGGCTCCCCGAAGAAGAAGAGGAAGGTGTGAagatcttaaagcggccgcccggctgcagatcgttcaaacatttggcaataaagtttcttaagattgaatcctgttgccggtcttgcgatgattatcatataatttctgttgaattacgttaagcatgtaataattaacatgtaatgcatgacgttatttatgagatgggtttttatgattagagtcccgcaattatacatttaatacgcgatagaaaacaaaatatagcgcgcaaactaggataaattatcgcgcgcggtgtcatctatgttactagatcggattcgtaatcatggtcatagctgtttcctg

**>Sequence of d35S-Intein^C^-P3-Intein^N^-Nos**

The d35S, intein^c^ and intein^N^ were shadowed green, purple and yellow, respectively. The sequence of P3 are in red, the terminator was shadowed grey.

gttgtaaaacgacggccagtgccaagcttgattaccaattcgagctccctacccctactccaaaaatgtcaaagatacagtctcagaagaccaaagggctattgagacttttcaacaaagggtaatttcgggaaacctcctcggattccattgcccagctatctgtcacttcatcgaaaggacagtagaaaaggaaggtggctcctacaaatgccatcattgcgataaaggaaaggctatcattcaagatgcctctgccgacagtggtcccaaagatggacccccacccacgaggagcatcgtggaaaaagaagacgttccaaccacgtcttcaaagcaagtggattgatgtgacatctccactgacgtaagggatgacgcacaatcccacccctactccaaaaatgtcaaagatacagtctcagaagaccaaagggctattgagacttttcaacaaagggtaatttcgggaaacctcctcggattccattgcccagctatctgtcacttcatcgaaaggacagtagaaaaggaaggtggctcctacaaatgccatcattgcgataaaggaaaggctatcattcaagatgcctctgccgacagtggtcccaaagatggacccccacccacgaggagcatcgtggaaaaagaagacgttccaaccacgtcttcaaagcaagtggattgatgtgacatctccactgacgtaagggatgacgcacaatcccactatccttcgcaagacccttcctctatataaggaagttcatttcatttggagaggacagcccagatcactagtATGATCAAGATCGCCACAAGGAAGTACCTCGGCAAGCAGAACGTGTACGATATCGGCGTGGAGCGCGACCACAACTTCGCCCTCAAGAATGGCTTCATCGCCTCCAACTGCCTCTACAGCCTCGAGGCCATCCCGCTCGAGGATCTGCTCAACAACCCGTTCAACTACGAGGTGGACCACATCATCCCACGCTCCGTGTCCTTCGACAACTCTTTCAACAACAAGGTTCTCGTGAAGCAGGAGGAGAATAGCAAGAAGGGCAACAGGACCCCGTTCCAGTACCTCTCCAGCTCCGACTCCAAGATCTCATACGAGACCTTCAAGAAGCACATCCTCAACCTCGCCAAGGGCAAGGGCAGGATCTCTAAGACCAAGAAGGAGTACCTCCTGGAGGAGAGGGACATCAACAGGTTCAGCGTGCAGAAGGATTTCATTAACCGCAACCTGGTGGACACCCGGTACGCGACCAGGGGCTTGATGAACCTCCTCAGGTCCTACTTCCGCGTGAACAACCTGGATGTGAAGGTGAAGTCCATCAACGGCGGCTTCACCAGCTTCCTCCGCCGGAAGTGGAAGTTCAAGAAGGAGCGCAACAAGGGCTACAAGCACCACGCCGAGGATGCCCTGATCATCGCGAACGCCGACTTCATCTTCAAGGAGTGGAAGAAGCTGGACAAGGCCAAGAAAGTGATGGAGAATCAGATGTTCGAGGAGAAGCAGGCCGAGTCAATGCCTGAGATCGAGACCGAGCAGGAGTACAAGGAGATCTTCATCACCCCGCACCAGATCAAGCACATCAAGGACTTCAAGGATTACAAGTACTCCCACAGGGTGGATAAGAAGCCGAATCGCGAGCTGATCAACGATACCCTGTACTCCACACGCAAGGACGACAAGGGCAACTGCCTCTCCTACGAGACCGAGATCCTCACAGTGGAGTACGGCCTCCTCCCGATCGGCAAGATCGTGGAGAAGCGGATCGAGTGCACCGTGTACAGCGTCGATAACAACGGCAACATCTACACCCAGCCGGTGGCCCAGTGGCATGATCGCGGCGAGCAGGAGGTGTTCGAGTACTGCCTCGAGGACGGCTCACTCATCCGCGCCACAAAGGACCACAAGTTCATGACCGTGGACGGCCAGATGCTCCCAATCGATGAGATCTTCGAGAGGGAGCTGGACCTCATGAGGGTGGATAACCTCCCGAACTGAagatcttaaagcggccgcccggctgcagatcgttcaaacatttggcaataaagtttcttaagattgaatcctgttgccggtcttgcgatgattatcatataatttctgttgaattacgttaagcatgtaataattaacatgtaatgcatgacgttatttatgagatgggtttttatgattagagtcccgcaattatacatttaatacgcgatagaaaacaaaatatagcgcgcaaactaggataaattatcgcgcgcggtgtcatctatgttactagatcggattcgtaatcatggtcatagctgtttcctg

**>Sequence of d35S-Intein^C^-P4-Nos**

The d35S and intein^C^ were shadowed green and purple, respectively. The sequence of P4 and NlS are in red and green, respectively. The terminator was shadowed grey.

gttgtaaaacgacggccagtgccaagcttgattaccaattcgagctccctacccctactccaaaaatgtcaaagatacagtctcagaagaccaaagggctattgagacttttcaacaaagggtaatttcgggaaacctcctcggattccattgcccagctatctgtcacttcatcgaaaggacagtagaaaaggaaggtggctcctacaaatgccatcattgcgataaaggaaaggctatcattcaagatgcctctgccgacagtggtcccaaagatggacccccacccacgaggagcatcgtggaaaaagaagacgttccaaccacgtcttcaaagcaagtggattgatgtgacatctccactgacgtaagggatgacgcacaatcccacccctactccaaaaatgtcaaagatacagtctcagaagaccaaagggctattgagacttttcaacaaagggtaatttcgggaaacctcctcggattccattgcccagctatctgtcacttcatcgaaaggacagtagaaaaggaaggtggctcctacaaatgccatcattgcgataaaggaaaggctatcattcaagatgcctctgccgacagtggtcccaaagatggacccccacccacgaggagcatcgtggaaaaagaagacgttccaaccacgtcttcaaagcaagtggattgatgtgacatctccactgacgtaagggatgacgcacaatcccactatccttcgcaagacccttcctctatataaggaagttcatttcatttggagaggacagcccagatcactagtATGATCAAGATCGCCACAAGGAAGTACCTCGGCAAGCAGAACGTGTACGATATCGGCGTGGAGCGCGACCACAACTTCGCCCTCAAGAATGGCTTCATCGCCTCCAACTGCCTCTACAGCCTCGAGGCCATCCCGCTCGAGGATCTGCTCAACAACCCGTTCAACTACGAGGTGGACCACATCATCCCACGCTCCGTGTCCTTCGACAACTCTTTCAACAACAAGGTTCTCGTGAAGCAGGAGGAGAATAGCAAGAAGGGCAACAGGACCCCGTTCCAGTACCTCTCCAGCTCCGACTCCAAGATCTCATACGAGACCTTCAAGAAGCACATCCTCAACCTCGCCAAGGGCAAGGGCAGGATCTCTAAGACCAAGAAGGAGTACCTCCTGGAGGAGAGGGACATCAACAGGTTCAGCGTGCAGAAGGATTTCATTAACCGCAACCTGGTGGACACCCGGTACGCGACCAGGGGCTTGATGAACCTCCTCAGGTCCTACTTCCGCGTGAACAACCTGGATGTGAAGGTGAAGTCCATCAACGGCGGCTTCACCAGCTTCCTCCGCCGGAAGTGGAAGTTCAAGAAGGAGCGCAACAAGGGCTACAAGCACCACGCCGAGGATGCCCTGATCATCGCGAACGCCGACTTCATCTTCAAGGAGTGGAAGAAGCTGGACAAGGCCAAGAAAGTGATGGAGAATCAGATGTTCGAGGAGAAGCAGGCCGAGTCAATGCCTGAGATCGAGACCGAGCAGGAGTACAAGGAGATCTTCATCACCCCGCACCAGATCAAGCACATCAAGGACTTCAAGGATTACAAGTACTCCCACAGGGTGGATAAGAAGCCGAATCGCGAGCTGATCAACGATACCCTGTACTCCACACGCAAGGACGACAAGGGCAACACCCTCATCGTGAACAACCTCAACGGCCTCTACGACAAGGACAACGACAAGCTCAAGAAGCTCATCAACAAGTCCCCGGAGAAGCTCCTCATGTACCACCACGATCCGCAGACCTACCAGAAGCTCAAGCTCATCATGGAGCAGTACGGCGATGAGAAGAACCCACTCTACAAGTACTACGAGGAGACCGGCAACTATCTCACCAAGTACTCTAAGAAGGACAACGGCCCAGTGATCAAGAAGATCAAGTACTACGGCAATAAGCTCAACGCCCACCTCGACATCACCGACGATTACCCGAACTCCCGGAACAAGGTGGTCAAGCTGAGCCTGAAGCCGTACAGGTTCGACGTTTACCTCGATAATGGCGTGTACAAGTTCGTGACCGTGAAGAACCTTGATGTGATCAAGAAGGAGAACTACTACGAGGTGAACAGCAAGTGCTACGAGGAGGCCAAGAAGCTGAAGAAGATCAGCAACCAGGCCGAGTTCATCGCGAGCTTCTACAACAACGATCTCATCAAGATCAACGGCGAGCTCTACCGCGTGATCGGCGTCAACAACGATCTCCTCAACCGCATCGAGGTGAACATGATCGACATCACCTACCGCGAGTACCTCGAGAACATGAACGACAAGAGGCCGCCGCGCATCATCAAGACCATCGCCAGCAAGACCCAGTCCATCAAGAAGTACTCCACCGACATCCTCGGCAACCTCTACGAGGTGAAGTCCAAGAAGCACCCGCAGATCATCAAGAAGGGCTCCGGCGGCAGCCCGAAGAAGAAGCGCAAGGTGAGCGGCGGCTCCCCTAAGAAGAAGCGCAAGGTGAGCGGCGGCTCCCCGAAGAAGAAGAGGAAGGTGTGAagatcttaaagcggccgcccggctgcagatcgttcaaacatttggcaataaagtttcttaagattgaatcctgttgccggtcttgcgatgattatcatataatttctgttgaattacgttaagcatgtaataattaacatgtaatgcatgacgttatttatgagatgggtttttatgattagagtcccgcaattatacatttaatacgcgatagaaaacaaaatatagcgcgcaaactaggataaattatcgcgcgcggtgtcatctatgttactagatcggattcgtaatcatggtcatagctgtttcctg

**>Sequence of OsU3-gRNA**

The OsU3 promoter and gRNA scaffold were shadowed yellow and green, respectively.

taattcatccaggtctccaagttctaggattttcagaactgcaacttattttatcaaggaatctttaaacatacgaacagatcacttaaagttcttctgaagcaacttaaagttatcaggcatgcatggatcttggaggaatcagatgtgcagtcagggaccatagcacaagacaggcgtcttctactggtgctaccagcaaatgctggaagccgggaacactgggtacgttggaaaccacgtgatgtgaagaagtaagataaactgtaggagaaaagcatttcgtagtgggccatgaagcctttcaggacatgtattgcagtatgggccggcccattacgcaattggacgacaacaaagactagtattagtaccacctcggctatccacatagatcaaagctgatttaaaagagttgtgcagatgatccgtggcaGCTCGCAGGTGAACACAACACCTGCACACGTTTTAGTACTCTGTAATTTTAGGTATGAGGTAGACGAAAATTGTACTTATACCTAAAATTACAGAATCTACTAAAACAAGGCAAAATGCCGTGTTTATCTCGTCAACTTGTTGGCGAGATTTTTTT

**Supplementary table 1. Primer sets used in this study**

| **Name** | **Sequence 5'-3'** | **Description** |
| --- | --- | --- |
| U3pmeF | CCTGTCAAACACTGATAGTTTgtaattcatccaggtctccaag | Construct the gRNA expression cassette and clone it into the Pme I restriction site. |
| U3pmeR | GTCGTTTCCCGCCTTCAGTTTaaaaaaatctcgccaacaagttg |  |
| OsNYC1-T1F | AGGCGGCGATTCAGGGGCTCGGTTTTAGTACTCTGTAAT | Generate a target site for editing the rice *OsNYC1* gene. The target sequences are in red. |
| OsNYC1R-T1R | CGAGCCCCTGAATCGCCGCCTtgccacggatcatctgcacaa |  |
| OsNYC4F-T1F | CCGTCTATGACCAACTCATGGGTTTTAGTACTCTGTAAT | Generate a target site for editing the rice *OsNYC4* gene.The target sequences are in red. |
| OsNYC4R-T1R | CCATGAGTTGGTCATAGACGGtgccacggatcatctgcacaa |  |
| OsPDSF-T1F | AAACCCATATTGCTTGAGGCAGTTTTAGTACTCTGTAAT | Generate a target site for editing the rice *OsPDS* gene.The target sequences are in red. |
| OsPDSR-T1R | TGCCTCAAGCAATATGGGTTTtgccacggatcatctgcacaa |  |
| t-OsNYC1F | CCGCGGTGGTCCACCTCTCC | Identify the editing types of the *OsNYC1* gene. |
| t-OsNYC1R | GCGCTCGCTAACGCATTGAGTG |  |
| t-OsNYC4F | TTATAACATATTGTACACAGCATGA | Identify the editing types of the *OsNYC4* gene. |
| t-OsNYC4R | ACTTGGATCAATTAAGTGTAGGA |  |
| t-OsPDSF | CTAAACCATTACAGGTCGTGATT | Identify the editing types of the *OsPDS* gene. |
| t-OsPDSR | CCTTAGAGATATCTATCAGTGC |  |
| Full-F | TTTATTGCCAAATGTTTGAACGATCGGGGAAATTCGGATCCCCAATACTTCAC | Clone the full-length SaCas9 into the vector. |
| Full-R | TTGTTTGGTGTTACTTCTGCAGCCCGGGGGATCCCCAATACTATGGCC |  |
| v1-P1-F | tgttacttctgcagcccgggggatccATGAAGCGCAACTACATCCT | Clone the P1-Intein^N^ of split-v1 into the vector. |
| v1-P1-R | ttgaacgatcggggaaattcggATCTCAGTTCGGGAGGTTATCCAC |  |
| v1-P2-F | gacagcccagatcactagtATGATCAAGATCGCCACAA | Clone the Intein^C^-P2 of split-v1 into the vector. |
| v1-P2-R | cgggcggccgctttaagatctTCACACCTTCCTCTTCTTCT |  |
| v2-P1-R | ttgaacgatcggggaaattcggATCTCACTTGCCCTCCTGCATATCAT | Clone the P1 of split-v2 into the vector. |
| v2-P2-F | gacagcccagatcacTGCCTCTACAGCCTCGAGGCC | Clone the P2 of split-v2 into the vector. |
| v3-P3P4-F1 | agttcatttcatttggagaggacagcccagatcactagtATGATCAAGATCGCCAC | Clone the Intein^N^-P3-Intein^C^ or Intein^N^-P4 of split-v3 into the vector. |
| v3-P3-R1 | ccgggcggccgctttaagatctTCAGTTCGGGAGGTTATCCACCC |  |
| v3-P4-R1 | gaaactttattgccaaatgtttgaacgatctgcagccgggcggccgctttaagatct |  |
| γ1SaNF | ATTTACCTTCGCtaaggaaGTTTAAATGAAGCGCAACTACATCCTCG | Clone the P1-Intein^N^ of split-v1 into the γ1. |
| γ1SaNR | TTAaccaccaccaccgtTCAGTTCGGGAGGTTATCCA |  |
| γ1SaCF | ATTTACCTTCGCtaaggaaGTTTAAATGATCAAGATCGCCACAAGG | Clone the Intein^C^-P2 of split-v1 into the γ1. |
| γ1SaCR | TTAaccaccaccaccgtTCACACCTTCCTCTTCTTC |  |
| γ2SaNF | ACACTTCGGCtaaggaAGTTTAAATGAAGCGCAACTACATCCTCG | Clone the P1-Intein^N^ of split-v1 into the γ2. |
| γ2SaNR | ggccagccaccgccaccagtTCAGTTCGGGAGGTTATCCA |  |
| γ2SaCF | ACACTTCGGCtaaggaAGTTTAAATGATCAAGATCGCCACAAGG | Clone the Intein^C^-P2 of split-v1 into the γ2. |
| γ2SaCR | ggccagccaccgccaccagtTCACACCTTCCTCTTCTTC |  |
| γ1-LcHRC-T1F | ATTTACCTTCGCtaaggaaGGGAGGCGAAGCTATTCTTCCTgttttagtactCTGGAAa | Clone sgRNA targeting *LcHRC, LcGW2 and LcTB1* gene into the γ1. The target sequences are in red. |
| γ1-LcHRC-T2F | ATTTACCTTCGCtaaggaaGGGTAGCGGCGCCTGCGGCTCCgttttagtactCTGGAAa |  |
| γ1-LcTB1-T1F | ATTTACCTTCGCtaaggaaGCGTCGCCGGCGAATCCAGGGGgttttagtactCTGGAAa |  |
| γ1-LcGW2-T1F | ATTTACCTTCGCtaaggaaGCAACAGCACAAGAAAATCCACgttttagtactCTGGAAa |  |
| γ1-LcGW2-T2F | ATTTACCTTCGCtaaggaaGGGTGGAAGCATGTGTGCTGTGgttttagtactCTGGAAa |  |
| γ1-gRNAR | TTAaccaccaccaccgtaaaaaaatctcgccaacaagttg |  |
| Hi-HRC-T1F | ggagtgagtacggtgtgcAACTGGAAGCAGCAATTAAGGCC | Detect mutations at the LcHRC-T1 site. |
| Hi-HRC-T1R | gagttggatgctggatggCCATCAGAGTCAGAGTGGCCATG |  |
| Hi-LcHRC-T2F | ggagtgagtacggtgtgcCAACCAGGAAGGACCATTC | Detect mutations at the LcHRC-T2 site. |
| Hi-LcHRC-T2R | gagttggatgctggatggGACCCCTTGCCATCATTCATC |  |
| Hi-LcGW2-T1F | ggagtgagtacggtgtgcATACCCCATCCCGGTGG | Detect mutations at the LcGW2-T1 and LcGW2-T2 sites. |
| Hi-LcGW2-T2F | ggagtgagtacggtgtgcCATTGCTGGTGGAAGCA |  |
| Hi-LcGW2-T1T2R | gagttggatgctggatggGTTCTACCATGAGCTTCTGC |  |
| Hi-LcTB1-T1F | ggagtgagtacggtgtgcGGGGCTCTGGAACCATT | Detect mutations at the LcTB1-T1 site. |
| Hi-LcTB1-T1R | gagttggatgctggatggTGTTGGAAGGCGTCGCC |  |
